# Piriformis Syndrome in Pre-monsoon, Monsoon, and Winter: An Observational Pilot Study

**DOI:** 10.7759/cureus.35296

**Published:** 2023-02-22

**Authors:** Md. Abu B Siddiq, Md. Shawkat Hossain, Amin Uddin A Khan, Md. Abu Sayed, Johannes J Rasker

**Affiliations:** 1 Physical Medicine and Rehabilitation, Brahmanbaria Medical College, Brahmanbaria, BGD; 2 Rheumatology, University of South Wales, Pontypridd, GBR; 3 Physical Medicine and Rehabilitation, Chittagong Medical College, Chittagong, BGD; 4 Physical Medicine and Rehabilitation, Marine City Medical College, Chittagong, BGD; 5 Community Medicine, Brahmanbaria Medical College, Brahmanbaria, BGD; 6 Rheumatology, University of Twente, Enschede, NLD

**Keywords:** seasonal, rheumatic disease, gluteal pain, low back pain, seasons, piriformis syndorme

## Abstract

Introduction: Piriformis syndrome (PS) is a rare focal soft tissue rheumatic disease. Due to heavy rural work, we questioned whether PS was more prevalent in the rainy monsoon than in other seasons. In this pilot research, we studied the pattern of PS, the frequency of PS over the seasons, and whether there were typical preceding events.

Methods: In this time-series descriptive study, PS cases diagnosed in a community-based clinic between January 2018 and December 2019 were enrolled. PS was diagnosed by clinical features and a 50% immediate pain relief from ultrasonogram-guided lidocaine (2%) injection in the piriformis muscle (PM). PS mimics were excluded.

Results: A total of 38 PS cases (11 males) were enrolled consecutively. In 2018, during dry winter (November-February), pre-monsoon (March-May), and rainy monsoon (June-October), nine, seven, and one PS cases were diagnosed, respectively; in 2019, the numbers were three, eight, and seven, respectively. Thus, over two years, 12 PS patients were diagnosed in dry winter, 15 in pre-monsoon, and eight in rainy monsoon. There was no correlation with the type of preceding events. There were no differences in the pattern of PS between the seasons.

Conclusions: In this pilot study, over two years more new PS cases were observed in the pre-monsoon and dry winter than in the rainy season; this was not supporting our research question. There was no association with specific preceding events.

## Introduction

Piriformis syndrome (PS) was first named by Robinson [1]. PS or 'deep gluteal syndrome' is an extra-spinal compressive sciatic nerve (SN) neuropathy with piriformis muscle (PM) spasm [2]. The clinical presentation of PS is inconsistent; sometimes, it is a deep-seated localized gluteal pain due to predominate PM, and sometimes, the PM spasm irritates the SN vicinity and generates radiating pain [3,4]. In PS, long-time sitting increases deep-gluteal pain [3]. Ambulation improves pain in chronic cases; however, in some patients, discomfort may increase on walking [4]. PS is more prevalent in women due to the wide Q-angle of the pelvis [3]. PS is a disorder of exclusion with no diagnostic or classification criteria [5]; however, Hopayian et al. described standard diagnostic features and tests in PS [6]: 

PS may be mistaken as wallet neuritis, lumbago sciatica, prolapsed lumbar intervertebral disc (PLID), piriformis pyomyositis, lotus neuropathy, quadratus lumborum, gluteal medius myofascial pain syndrome (MPS), osteitis condensans illi, spondyloarthropathy (SpA), post-injection sciatica, and sciatic nerve tumor [1]. When researching PS over the years [1,3,4], we got the impression that the incidence might be unevenly spread over the year and seasons. There was no data on how PS is associated with weather changes. Bangladesh is a delta full of rivers and people are engaged in fishing, manual jobs, and agriculture with increasing crops over the last few years [7]. In general, paddy is planted and harvested manually. These physical activities involve the lower back and gluteal structures, including PM and sciatic nerve [3,4,7].

We expected that the incidence of PS would be highest in the rainy monsoon, due to heavy work and frequent falling. We also wondered whether the incidence of PS changed over the seasons and whether there was an association with preceding events. Hence, we decided to explore the issue in an observational pilot study. For that reason, we decided to study whether the incidence of PS varies over the three main seasons. If that was the case, it would be of help to the physicians in charge of this rare condition.

## Materials and methods

Study participants and diagnosis of PS

**Figure 1:**
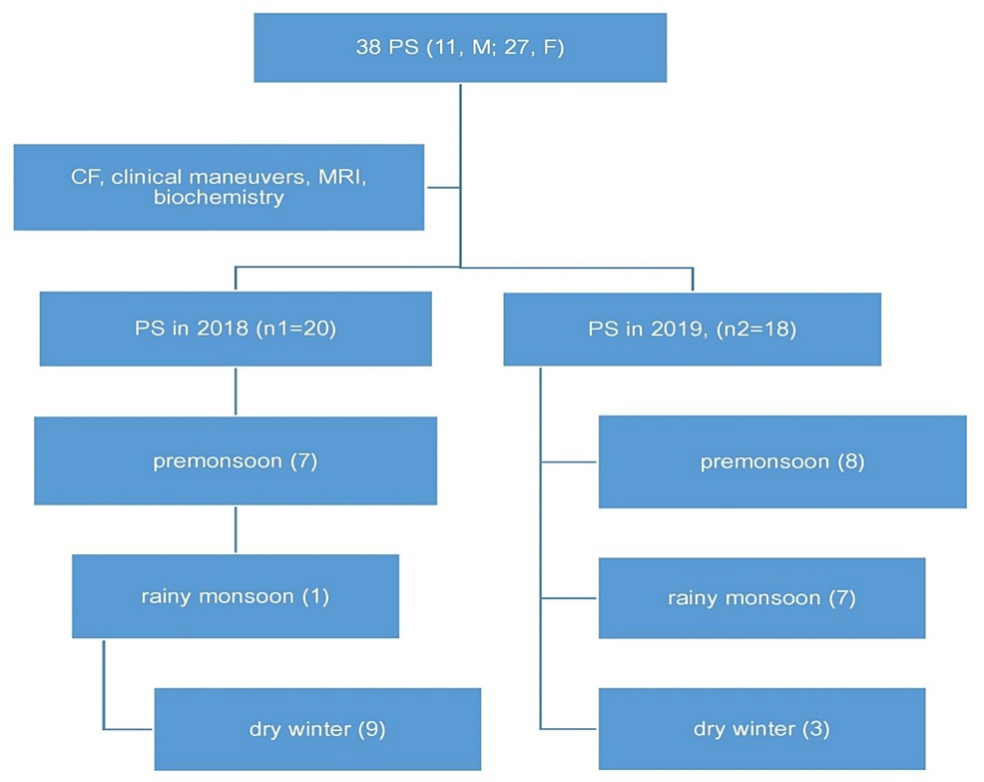
Study participants PS, Piriformis Syndrome; F, Female; M, Male; CF, Clinical Feature; MRI, Magnetic Resonance  Imaging

The study was conducted per the Helsinki Declaration of 1975, as revised in 1983. The Chittagong Medical College Ethical Review Committee approved the work (approval number: CMC/PG/2014/5). Patients' consent was taken. This descriptive observational pilot study of PS was performed over two years at a government-registered community-based clinic named Feni Clinic, Feni, Bangladesh, from January 2018 to December 2019 (Figure 1).

In the study, we diagnosed the PS according to Hopayian et al. [6]. These include clinical features, buttock pain, external tenderness over the greater sciatic notch, gluteal pain aggravated through sitting, and some maneuvers: the flexion-adduction-internal rotation (FAIR) test, the Pace sign, Freiberg test, and digital rectal examination [8]. We also performed musculoskeletal ultrasonography (MSUS)-guided lidocaine (2%) 10 mL) injection; in PM at least a 50% reduction of pain intensity favored PS diagnosis [3].

The Freiberg test measures gluteal pain during passive internal rotation of the extended hip. The Pace sign shows sciatic symptoms when the patient is asked to abduct the affected hip against resistance while remaining seated [8]. The FAIR test is the most sensitive and widely used method to classify PS [8]. During the maneuver, the patient remains supine, keeping the affected hip and knee flexed at 60 degrees and 90 degrees, respectively, with an internally rotating and adducting hip joint. The FAIR test is associated with Lasègue's sign in the modified FAIR test [8]. During the internal rectal examination, while fingers glide over the lateral pelvic wall deep gluteal tenderness may indicate PS as the cause of the complaints [3]. Gaenslen's test and straight leg raise (SLR) test were also done to exclude inflammatory sacroiliitis and lumbar nerve root compression at the spine level, respectively [3]. The principal researcher (Md. ABS) took the history, examined all patients, and performed all clinical maneuvers.

X-rays and magnetic resonance imaging (MRI) of the lumbosacral spine, hip, pelvis, sacroiliac, and hip joints were performed to exclude clinical mimics. Laboratory tests included C-reactive protein (CRP) and erythrocyte sedimentation rate (ESR) to exclude infectious origin of the complaints, for example, in piriformis pyomyositis [9].

Data collection and outcome variables

Data were recorded in a pre-formed data sheet. The primary outcome variable was the number of PS cases over the study period. Among six various seasons, only three of them are distinguishable in the country of the recent study: (i) the pre-monsoon (March through May), (ii) rainy monsoon (June through October), and (iii) dry winter (November through February) [7]. We registered the number of PS cases in these three seasons. Preceding events in PS were recorded, such as loading-unloading, a sudden shift in body position (standing from long-time sitting), preparing paddy, recent falls, lifting weights, blunt trauma over lower back region (LBR), and anemia.

Statistics and data presentation

**Figure 2:**
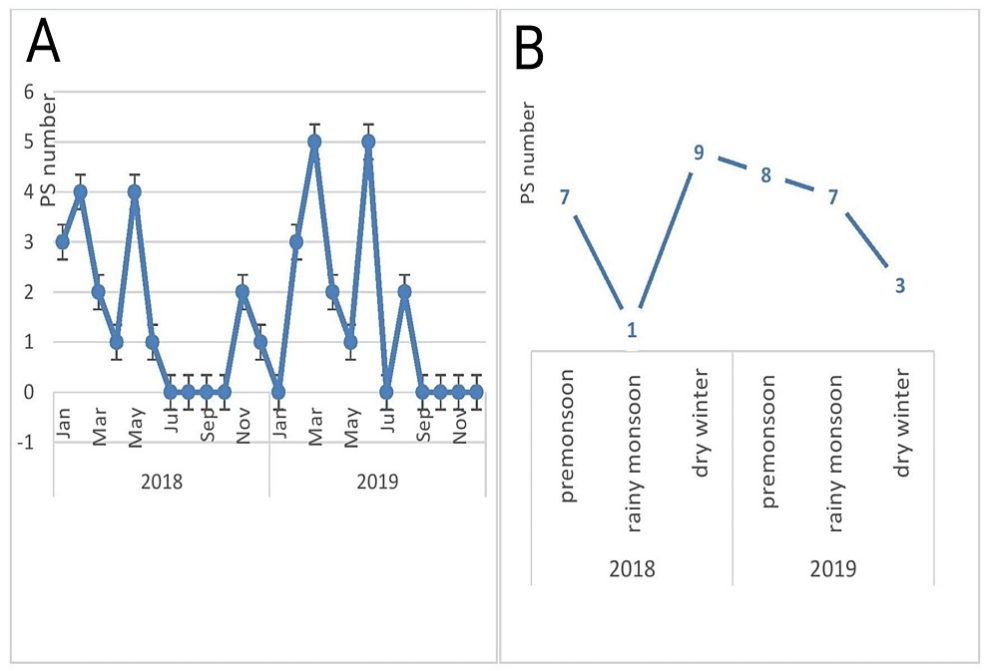
Piriformis syndrome in seasons: (A) number of PS over months, (B) PS pattern over seasons PS, Piriformis Syndrome; Jan, January; Mar, March; Jul, July; Sep, September; Nov, November

Quantitative variables were expressed in frequency, means, and standard deviations (SD); qualitative data were expressed in percentage ratios. Data were analyzed using IBM SPSS Statistics for Windows, Version 25.0 (Released 2017; IBM Corp., Armonk, New York, United States. Missing data were handled in SPSS using a roman number in the list. The line graph was made using Microsoft Office Excel (2016; Microsoft Corporation, Redmond, Washington, United States). PS frequency was recorded and plotted in a line graph (Figure 2). 

## Results

From January 2018 to December 2019, 38 PS (18 PS in each year; in 2018, one patient developed right and left PS at a few months interval) cases were registered (male 11) in a community clinic. In 2018 and 2019, the mean age at presentation for PS cases was 45.26 ± 6.38 and 43.5 ± 7.48 years (p, 0.436 at 95% CI), respectively. The mean body mass index (BMI) was 22.72 ± 2.39 and 22.99 ± 3.87 (p, 0.795 at 95% CI), respectively. The demographic data are summarized in Table 1. 

**Table 1:** Demography of study participants *p>0.05; **day laborer, wood chopper; ***elderly people, living abroad BMI, Body Mass Index; PS, Piriformis Syndrome

Characteristics	2018 (n =20)	2019 (n=18)
Sex (male / female) (n)	6/14	5/13
Age at presentation (years ± SD)*	45.26 ± 6.38	43.5 ± 7.48
Involved side (n) (right / left)	5/15	9/9
BMI*	22.72 ± 2.39	22.99 ± 3.87
PS in rural area	11	10
PS in urban area	07	08
Occupations		
Housewives	09	12
Cleaner	01	00
Electrician	01	00
Scavengers	01	00
Manual workers^**^	01	01
Not specified^***^	05	04
Comorbidity		
Leg-length discrepancy	02	00
Fibromyalgia Syndrome	00	01
Prolapsed lumbar intervertebral disc (PLID) & lumbar ligament sprain	00	01
Lumbar spinal stenosis	00	04

Table 2 depicts the clinical characteristics of PS. 

**Table 2:** Clinical presentations of piriformis syndrome *One patient presents with right-left shifting of PS manifestations, **Five PS cases in association with lumbar spinal stenosis Pace sign was negativity FAIR: Flexion-Adduction-Internal Rotation

Characteristics	2018	2019
Gluteal pain	20 (100%)	18 (100%)
Pain aggravated sitting	20 (100%)	18 (100%)
Pain with tingling in the lower limb	17 (85%)	9 (50%)
Pain on squatting	20 (100%)	18 (100%)
Pain reduced with walking	14 (70%)	9 (50%)
Deep gluteal tenderness	20 (100%)	18 (100%)
FAIR test	20 (100%)	18 (100%)
Pace sign	18 (90%)	13 (72.2%)**
Freiberg test	20 (100)	18 (100%)
Straight leg raising test positive	5 (25%)	2 (11.11%)
Digital per-rectal examination	20 (100%)	18 (100%)

Gluteal pain was the most frequent complaint among study subjects. In 2018, increased pain affected side sitting (>15 minutes), forward bending, and kneeling-squatting were documented in 20, 19, and 20 cases, respectively, whereas in 2019, the figures were 18, 8, and 18, respectively. Tingling in the respective lower limb was documented in 26 PS patients in both years. Walking improved gluteal pain in 14 and 18 PS patients for the years 2018 and 2019, respectively. Physical maneuvers that were found positive in PS patients were: FAIR (38), Pace sign (30), Freiberg test (38), and per rectal examination (38). SLR maneuvers were positive in all (eight in both years) acute PS cases. SLR and Pace signs were negative in all four PS cases associated with lumbar spinal stenosis. The Pace sign was also negative in four young primary PS patients. The pattern of PS appears not to differ over the seasons.

In 24 out of the 38 cases, there was no history of any preceding event. However, an initial fall, overuse of gluteal muscles (including PM), leg-length discrepancy (LLD), and fibromyalgia syndrome (FMS) were revealed in nine, two, two, and one cases, respectively. In one, PS improved on one side and three months later he developed PS on the opposite side; the patient ascribed this to long-standing kneeling and squatting during harvesting paddy. One PS patient with severe anemia reported symptom improvement following blood transfusion (Table 3). 

**Table 3:** Preceding events with piriformis syndrome *In three cases no preceding event has been recorded; **severe anemia in association with PS symptoms, improved with blood transfusion LBR, Low Back Region; PS: Piriformis Syndrome

Characteristics	2018^*^	2019
Pre-monsoon (March, April, and May)		
Loading–unloading	0	0
While sudden standing from long-time sitting/preparing paddy	2	0
Recent fall/lifting weight from waist	1	0
Blunt trauma over LBR	1	0
Anemia	0	0
No event	3	8
Rainy monsoon (June, July, August, September, and October)		
Loading-unloading	0	0
While sudden standing from long-time sitting/preparing paddy	1	1
Recent fall/lifting weight from waist	0	1
Blunt trauma over LBR	0	0
Anemia	0	0
No event	0	1
Dry winter (November, December, January, and February)		
Loading-unloading	0	0
While sudden standing from long-time sitting/preparing paddy	1	0
Recent fall/lifting weight from waist	1	0
Blunt trauma over LBR	0	0
Anemia^**^	1	0
No event	6	3

During 2018, there were small peaks in January (four), May (four), and November (two) (Figure 2A). In 2018, information was missing in three cases regarding the date of presentation. In 2019, there were peaks in February (three), March (five), June (five), and August (two) (Figure 2A). The pattern of PS case distribution according to the seasons is presented in Figure 2B. Among the three seasons, 15 cases were recorded in the pre-monsoon, 12 were in dry winter, and eight were in the rainy monsoon. In 2018, there was a sharp fall in PS cases from pre-monsoon to rainy monsoon; then, it rose again sharply in the dry winter. However, in 2019, the pattern was different. In monsoon, PS frequency was the highest, and then it fell gradually during the rainy season. In dry winter, the PS frequency fell even more rapidly.

## Discussion

In the present study, we documented PS distribution in different seasons. Deep-seated gluteal pain and radiating sciatica-like complaints were the most presenting manifestations. All patients had deep gluteal tenderness and were positive for FAIR and Freiberg tests. Besides, ipsilateral gluteal tenderness was revealed on digital per-rectal examination. However, the Pace sign was negative in all PS with lumbar spinal stenosis. Higher PS frequency was reported in the pre-monsoon and dry winter than in the rainy monsoon.

PS is a rare cause of low back pain (LBP). The correlation between PS incidence and season changes has never been studied. In LBP, a few studies looked for an association with weather parameters [10]; however, most studies had limitations in the quality of the study design or relied on participants' subjective recall of weather, or were biased as patients were not blinded for the study hypothesis [10]. Hippocrates noted that seasons were linked with certain ailments and the Romans noted that cold and wet weather increased pain [11]. A study in Sydney found no association between the incidence of a new episode of acute LBP with precipitation, humidity, wind speed, wind gust, wind direction, and air pressure, but only with higher temperatures [12,13]. 

Temperature and vapor pressure may influence chronic or recurrent self-reported LBP [14]. In another study in England with chronic widespread pain (CWP), sufferers had the most pain in winter, intermediate in autumn and spring, and lowest in summer [15]. Fewer persons report pain on days with more hours of sunshine and when the average temperature was above 17.5 degrees Celsius [15]. However, this may be partly explained by the fact that on sunny and warmer days, these patients had more exercise, a better quality of sleep, and improved positive mood. [15].

The weather also influences non-LBP, for example, rheumatoid arthritis (RA) [16]. Patberg and Rasker described that RA variables positively correlate with the microclimate's humidity on the patient's skin [16]. High relative humidity is unfavorable but has less influence when there are few barriers to water vapor, like clothes, and when air conditioning is used. High temperature is unfavorable since it increases absolute humidity, but beneficial as well since it reduces the presence of barriers [16]. A prospective observational study with stable RA in Norway explored the association between joint pain and weather and solar variables [17]. The pain was associated with three or more external variables in 17% and one or two weather variables in 44% of cases. However, no associations were observed in 39% of the patients. There were consistently negative associations between pain, ultraviolet light dose, and solar radio flux/sunspot count [17]. A one-year-long study in Argentina revealed that low temperature, high atmospheric pressure, and high humidity significantly correlated with RA pain (p<0.001). In osteoarthritis (OA), the pain was associated with low temperature and high humidity (p < 0.001); however, in patients with FMS, pain correlated with low temperature and high atmospheric pressure (p < 0.001), while healthy controls did not show a correlation with bodily pain and weather changes [18]. Weather predominantly affects musculoskeletal symptoms in FMS, mainly in patients with weather beliefs [10].

In the current observational pilot study, during the first year, the incidence of PS appeared higher in the dry winter and pre-monsoon and less in the rainy monsoon, but in the second year, these findings were not confirmed. High temperatures and thunderstorms characterize the pre-monsoon with April being the hottest month [19]. We found that the number of new PS cases was higher in the hot and wet pre-monsoon, but this was not significant. Winter is the season of harvesting vegetables, with the general overuse of gluteal and pelvic musculature, including PM, and could induce PS [20,21].

There are two types of PS: primary and secondary to co-morbid conditions like lumbar spinal stenosis, leg-length discrepancy (LLD), myositis ossificans of PM, MPS of PM, and FMS [3,22]. Piriformis pyomyositis may be confused with PS and requires early diagnosis and definitive treatment [2,4,20,23,24]. In the present study, most cases (24/38) had primary PS; only 14 patients had associated lumbar spinal stenosis, LLD, and FMS. Overuse or spasm of PM during sudden posture change, preparing paddy with waist bending, fall, and blunt trauma over LBR were also reported. A patient with left PS may develop right-sided PS later, as documented in a male farmer. The patient claimed that rowing through a paddy field may have induced the second period. PM overuse and spasms can happen in prolonged sitting, planting paddy, running, and cycling [25]. Sometimes it may be associated with weak and tight hip abductor and adductor muscles [25].

Lumbago sciatica has reportedly been linked with PS, and double crush syndrome theory could also better explain PS in lumbar spinal stenosis [3]. Significant associations were reported between high cold exposure and regional and radiating LBP and should be recognized as a possible risk factor in occupational settings [26]. Irritation of trigger points that developed PM is supposed to aggravate PS in low-temperature in winter [26]. Poor ergonomics with poor body postures, including inappropriate postures during planting and harvesting crops, may develop trigger points in PM that may sensitize in cold and present with deep gluteal pain [3].

In an observational study of American adults aged over 65 years attending a total of 11,673,392 outpatient (OP) visits, no association was found between rainfall and the number of OP visits for any joint or back pain [16]. Rainfall in Bangladesh is copious during pre-monsoon, though less than in monsoon [20]; however, it may contribute to developing PS secondary to increased fall risk on slippery grounds. More understanding of the influence of temperature or season change on PS presentations is required [7]. Large-scale studies with multiple centers could reveal whether seasonal variations affect the disorder.

Study limitations and strengths

One limitation was that only one study center was involved. Second, we studied the frequency of PS only for two years; because of COVID-19, we could not include more patients after 2019. 

The strength of the study lies int he fact this is an extensive series of PS patients. This is the first study in the literature addressing the incidence and the pattern of PS as the season changes. It was performed in one center, so we likely have collected most cases from this region. 

## Conclusions

PS may present in a physician's office with deep-seated gluteal pain and may be confused with pain from the hip joint or with a herniated lumbar disc. We found an increased frequency of PS in pre-monsoon in both years; however, the pattern was not similar for other seasons. This difference may be incidental or because of environmental influence. More understanding of the influence of temperature or season change on PS presentations is required. Large-scale studies in multiple centers could reveal whether seasonal variations influence the incidence of new cases of PS. 
